# Dissecting the tumor microenvironment in response to immune checkpoint inhibitors via single-cell and spatial transcriptomics

**DOI:** 10.1007/s10585-023-10246-2

**Published:** 2023-12-08

**Authors:** Wendi Liu, Anusha Puri, Doris Fu, Lee Chen, Cassia Wang, Manolis Kellis, Jiekun Yang

**Affiliations:** 1 Computer Science and Artificial Intelligence Laboratory, Massachusetts Institute of Technology, Cambridge, MA USA; 2 Department of Stem Cell and Regenerative Biology, Harvard University, Cambridge, MA USA; 3 Department of Biological Engineering, Massachusetts Institute of Technology, Cambridge, MA USA; 4 Broad Institute of MIT and Harvard, Cambridge, MA USA

**Keywords:** Immunotherapy, Transcriptomics, Single-cell, Tumor microenvironment

## Abstract

Cancer is a disease that undergoes selective pressure to evolve during its progression, becoming increasingly heterogeneous. Tumoral heterogeneity can dictate therapeutic response. Transcriptomics can be used to uncover complexities in cancer and reveal phenotypic heterogeneity that affects disease response. This is especially pertinent in the immune microenvironment, which contains diverse populations of immune cells, and whose dynamic properties influence disease response. The recent development of immunotherapies has revolutionized cancer therapy, with response rates of up to 50% within certain cancers. However, despite advances in immune checkpoint blockade specifically, there remains a significant population of non-responders to these treatments. Transcriptomics can be used to profile immune and other cell populations following immune-checkpoint inhibitor (ICI) treatment, generate predictive biomarkers of resistance or response, assess immune effector function, and identify potential immune checkpoints. Single-cell RNA sequencing has offered insight into mRNA expression within the complex and heterogeneous tumor microenvironment at single-cell resolution. Spatial transcriptomics has enabled measurement of mRNA expression while adding locational context. Here, we review single-cell sequencing and spatial transcriptomic research investigating ICI response within a variety of cancer microenvironments.

## Introduction

Despite the fact that the field of cancer biology initially focused on studies of cancer cells, it has become evident that tumors are much more intricate than a simple aggregation of malignant cells. The tumor microenvironment (TME), which includes immune cells, extracellular matrix (ECM), blood vessels, and more, engages in constant interaction with cancer cells and creates the environment in which cancer cells habitate [1]. Because of such complexity, there is a growing recognition of the tumor and its microenvironment as an organ-like entity [2]. To better comprehend the TME, each specialized cell type, its contribution to the TME, and its specific response to therapy should be studied in detail. Here, we review the key cancer and immune interactions in the tumor and the single cell technologies that enabled these studies.

### Cancer immunity cycle

The human immune system constantly surveils the body to safeguard against developing cancer cells. The process of anti-tumor immune response can be summarized in the cancer immunity cycle [3]. To trigger the cancer immunity cycle, cancer cells at the tumor site generate neoantigens, which are frequently the results of non-synonymous somatic mutations, and present the neoantigens on cell surface through major histocompatibility complex (MHC). Antigen-presenting cells (APCs), such as dendritic cells (DCs), regularly sample antigens in the body and present fragments of the antigens through MHC I or MHC II. After capturing a cancer neoantigen and receiving signals from proinflammatory cytokines or cellular debris, APCs traffic to the lymph nodes, where they induce the expansion and differentiation of naive T cells into cytotoxic effector T cells [3]. The activated effector T cells migrate to the tumor site, recognize cancer cell peptide-MHCI (p-MHCI) through docking of the specific T cell receptor (TCR), and kill the cancer cells. The destruction of cancer cells during this process releases more cancer neoantigens and immunogenic signals, further reinforcing the cancer immunity cycle. However, this cancer immunity cycle could be interrupted if essential immune machinery or players are absent or impaired in the microenvironment. Deficiency in key immune cells in the cancer immunity cycle, often observed in individuals with HIV and patients undergoing pharmacological immunosuppression or organ transplant, can lead to higher cancer incidence, impaired cancer immunity, and poor response to immunotherapies [4].

### Tumor cells and immune evasion/immunoregulation

As roots of the cancer disease, cancer cells initiate the formation of tumors, shape the genetic characteristics of cancer, and drive the progression and metastasis of the disease [5]. The concepts of cancer stem cells (CSC) and circulating tumor cells (CTCs) have attracted attention in recent years. In certain solid cancer types, despite lacking fully validated biomarkers, CSCs are thought to constitute 0.05–1% of cancerous cells and possess remarkable abilities of self-renewal, differentiation, and tumorigenicity [6]. CSCs are often enriched in minimal residual disease and can promote therapeutic resistance and relapse [7]. Combination therapies such as chemotherapy and targeted therapy have been moderately efficacious in targeting CSCs [8]. On the other hand, CTCs emerge from the primary tumor and can be found in the bloodstream either as individual entities or clusters [6]. The presence of CTCs in the blood is actively exploited as biomarkers for diagnosis and treatment response, although the clinical utility remains controversial [9].

Beyond these two groups of cancer cells with special characteristics, the tumor’s composition is often marked by the presence of multiple clonal subpopulations of malignant cells arising from a combination of hyperproliferation and genome instability. This high intratumoral genetic heterogeneity contributes to the high degree of phenotypic diversity in response to therapy. As a result, treatments designed against a single specific mutation often fall short of completely eliminating all malignant cells and may inadvertently create selective pressure to promote the diversification of cancer cell populations [10]. This process of tumor evolution can lead to resistance to targeted therapy, resulting in the adoption of a regimen of several different treatments, each with an array of adverse effects. These therapeutic strategies necessitate the use of treatments like immunotherapy, which act directly on the immune system.

With a highly malleable genome, cancer cells have many ways to orchestrate the composition and functions of immune cells to achieve immune evasion, one of the cancer hallmarks [2, 5]. The genomic loss of the interferon gene cluster and epigenetic silencing of inflammatory mediators are direct ways to affect the immune response in the TME. For example, tumor cells can acquire resistance to therapy through loss of interferon (IFN)-ɣ pathway genes, which play a critical role in regulating T cell responses and tumor rejection [11]. On the other hand, oncogenic alterations, such as activation of RAS proteins, can also directly promote tumor associated inflammation, angiogenesis, and immunosuppression [12]. Oncogenic aberration of EGFR can promote regulatory T-cell (Treg) infiltration by upregulating CCL22 while hampering CD8 + T cell infiltration via IRF-1 mediated downregulation of CXCL10 [12]. The tumors may also avoid immune surveillance by presenting decreased tumor antigen or disrupting the antigen presentation system entirely. HER2 amplification downregulates MHC I expression and impairs interferon response by inhibiting the cGAS-STING pathway [12]. Case studies of melanoma and other cancers have reported acquired truncating mutations of B2M, a key gene in stabilizing MHC I molecules, at the time of progression [13]. Furthermore, cancer cells may hinder the cytotoxicity of infiltrating T and NK cells by secreting immunosuppressive factors such as TGF-β [14]. Cancer cells may also recruit immunosuppressive cells, such as Tregs and myeloid-derived suppressor cells (MDSCs) into TME to assist suppression of cytotoxic T cells. Moreover, tumors also exploit the natural ‘checkpoints’ on immune cells, putting a brake on their effector functions [15], which is the focus of this review.

### Immune checkpoints and immune checkpoint inhibitors (ICIs)

The combined action of co-stimulatory and co-inhibitory receptors regulates T cell activation. Alongside the APC presentation of peptide-MHC (p-MHC) to TCR, co-stimulatory receptor CD28 also plays a critical role in facilitating complete T cell activation. CD28 promotes T cell survival and proliferation by engaging with ligands of the B7 family on APCs. APCs also provide additional soluble factors and cytokines that activate the T cells to multiply and migrate to the target site. Complementing the co-stimulatory receptors are the co-inhibitory receptors known as immune checkpoint receptors (e.g. PD-1, LAG3, CTLA-4). These receptors serve as “checkpoints” to dampen T cell effector functions to prevent excessive inflammation in non-cancerous contexts. We will briefly go through the three major immune checkpoints with available ICIs in chronological order as they were approved by the FDA. ICIs represent a category within the broader field of immunotherapy, together with chimeric antigen receptor (CAR) T-cell therapy, etc. For the purposes of our discussion, the terms 'ICI', 'immunotherapy', and ‘immune therapy’ will be used interchangeably throughout this text.

The discoveries of checkpoint receptors, cytotoxic T-lymphocyte-associated protein-4 (CTLA-4) and programmed cell death protein 1 (PD-1), were groundbreaking achievements recognized by the 2018 Nobel Prize in Medicine [16]. CTLA-4 is expressed on the surface of activated T cells and has a high binding affinity to the B7 ligand, effectively out-competing CD28. By preventing the CD28-B7 engagement, CTLA-4 hinders the co-stimulatory signaling and suppresses T-cell-mediated anti-tumor immune responses. To counteract this mechanism, antibodies targeting CTLA-4 were developed to remove the CTLA-4 inhibitory signal and restore the antitumor effector function to T-cells. In 2011, ipilimumab (Yervoy) became the first ICI to receive FDA approval for metastatic melanoma. As of 2023, it remains the only CTLA-4 targeting ICI that received FDA approval [17].

T cells express PD-1, when under prolonged stimulation such as chronic viral infection or cancer [18]. Different from CTLA-4, which disrupts the CD28-B7 interaction, PD-1 operates at a more downstream stage of TCR signaling. The binding of PD-1 to its ligand PD-L1 (expressed on various immune, endothelial, and cancer cells) or PD-L2 (expressed on APCs and can be induced in cancer cells) triggers the recruitment of domain-containing phosphatase 1 (SHP1) and SHP2, which leads to dephosphorylation of the TCR complex [17]. Consequently, PD-1 signaling impedes T cell cytotoxicity function, cytokine secretion, and proliferation, and ultimately results in T cell exhaustion. To reinvigorate the antitumor activity of exhausted T cells, the FDA has approved three PD-1 blocking antibodies (nivolumab [Opdivo], pembrolizumab [Keytruda], and cemiplimab [Libtayo]) for various cancer indications [17, 19]. Additionally, three anti-PD-L1 blockades (atezolizumab [Tecentriq], durvalumab [Imfinzi], and avelumab [Bavencio]) working along the same pathway of action have been approved by FDA [20–22].

Anti-LAG3 (relatlimab) is the most recent FDA approved ICI after anti-CTLA-4 and anti-PD-1:PD-L1 antibodies. Lymphocyte activating gene 3 (LAG3) is a CD4 ancestral homolog and like CD4, LAG3 binds MHC II. As an immune checkpoint, LAG3 inhibits the activation of its host cell and promotes a suppressive immune response, reducing cytokine and granzyme production and encouraging differentiation into Tregs. Upon binding with MHC II or other LAG3 ligands (α-syn, Gal-3, LSECtin or FGL-1), LAG3’s cytoplasmic domain inhibits the early steps of the TCR pathway. As a result, activation of transcription factors (TFs) like NFAT is prevented, leading to a decrease in cytokine production and proliferation [23]. Although mainly studied on conventional T cells and Tregs, LAG3 is also expressed in various cells including unconventional T cells, NK cells, B cells, plasmacytoid DCs (pDCs), and neurons. Activated innate lymphoid cells can show significant levels of LAG3 expression, suggesting that LAG3 may have an important role in these cells [23]. However, the effects of LAG3-targeting on non-T cells have not been thoroughly studied, which is important to investigate as it may have a significant impact on their functions and the effectiveness of the therapy.

Due to the distinct T cell inhibition mechanisms of CTLA-4, PD-1, and LAG3, it was postulated that blocking multiple signals could enhance the reversal of exhausted T cell phenotype. As a result, combining ICI emerged as a logical treatment approach. In the clinical trial Checkmate 067, the efficacy of ICI combination targeting anti-CTLA-4 and anti-PD-1 was evaluated against single therapy in treatment naive melanoma patients [24]. The objective response rate was found to be 19.0% in the anti-CTLA-4 arm, 43.7% in the anti-PD-1 arm, and 57.6% in the combination arm [25]. The median overall survival (OS) was 19.9 months, 36.9 months, and over 60 months, respectively. While the combination therapy demonstrated higher clinical efficacy, it increased the incidence of grade 3 and 4 adverse events. Such adverse events can typically be managed by delaying treatment or administering corticosteroids systemically. Combined anti-LAG3 and anti-PD-1 therapy has also shown a strong antitumor effect in mice resistant to single-antibody treatment [26]. FDA has granted approval for the use of a combination of anti-PD-1 and anti-LAG3 in patients aged 12 or above who have previously untreated melanoma that is not amenable to surgical removal or has spread throughout the body. This decision was based on the outcomes of the RELATIVITY-047 clinical trial, which revealed that patients who received the combination therapy had a longer progression-free survival (PFS) of 13.2 months compared to patients who received anti-PD-1 alone (10.1 months) [27]. While the current understanding of these checkpoints is primarily in T cells, recent studies have identified the immune checkpoints in other cell types, which is reviewed in our back-to-back Review [28].

Undoubtedly, ICIs have revolutionized cancer treatment, leading to prolonged survival or even clinical “cure” for some patients. Despite advances in ICIs, there remains a significant population of non-responders to these treatments. The response rate can range from more than 70% (Classic Hodgkin Lymphoma) to nearly 50% (melanoma) to 15–30% (other solid tumors) [29, 30]. Without any highly sensitive biomarkers for ICI response prediction, the current practice of therapy assignment of ICIs is far from satisfactory, which is mostly based on clinical factors such as volume, site of malignancy, patient demography, and somatic mutation presence. Thus, it is urgent and of paramount significance to study ICI mechanisms in different cancer contexts using technologies with high throughput and high resolution. Single-cell technologies have emerged as potent and invaluable tools in propelling breakthroughs in this field.

## Understanding the tumor-immune microenvironment through single-cell technology

**Fig. 1:**
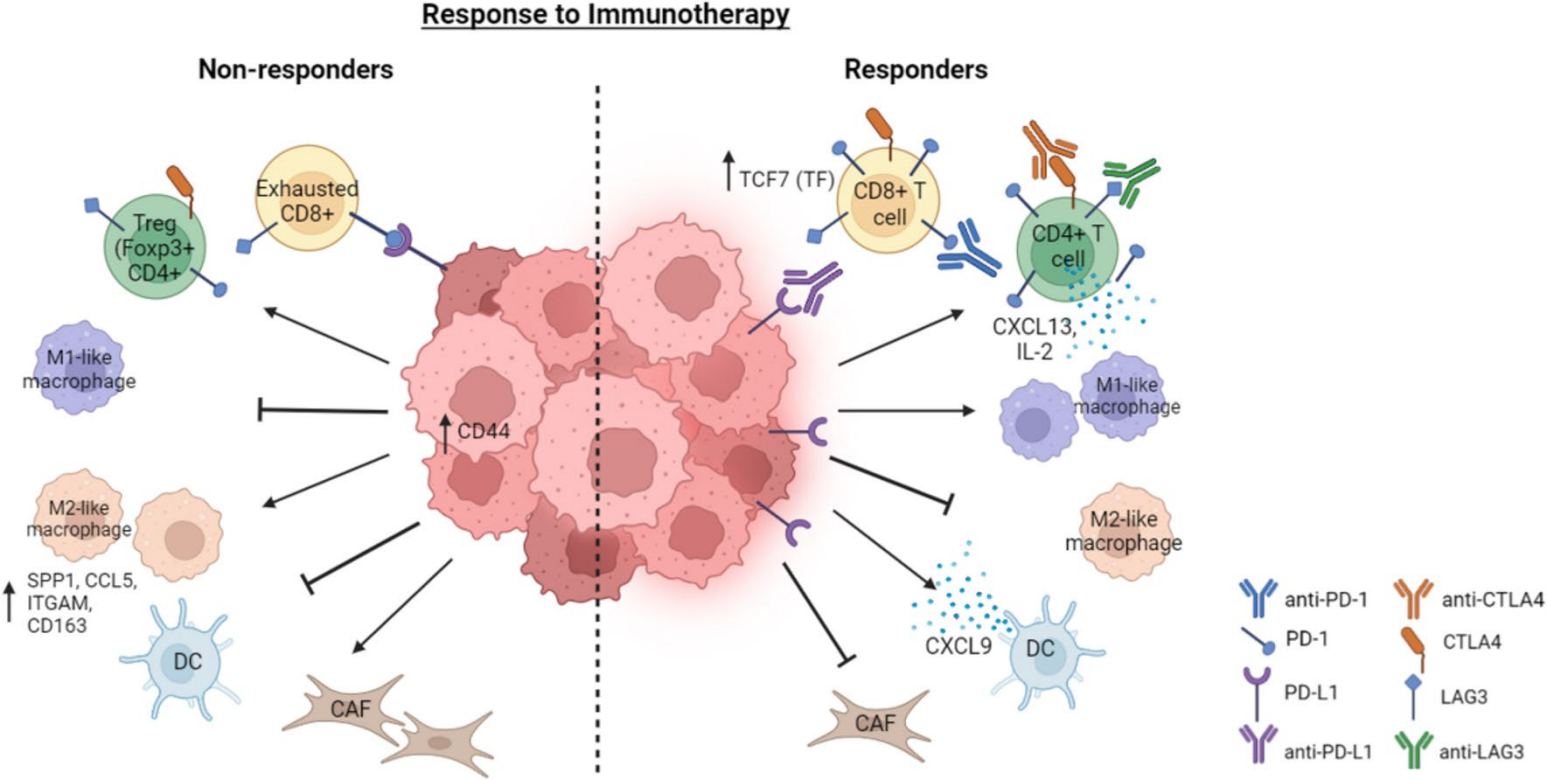
Cellular signaling and interaction in the tumor microenvironment (TME) in response to immune checkpoint inhibitors (ICIs). Adaptive and innate immune cell signaling within the TME of responders and non-responders to ICIs. Left: Increased CD44 expression promotes acquisition of cancer stem cell features and metastasis, alongside SPP1, CCL5, CD163, and ITGAM + macrophages. Reduced checkpoint expression on T cell surfaces and increased immunosuppressive molecule expression promotes refractory response to ICIs. Right: Decreased immunosuppressive cell abundance and secretion of anti-tumor effector molecules, coupled with increased checkpoint expression, renders tumors more responsive to ICIs. CAF, cancer-associated fibroblast; DC, dendritic cell; Treg, regulatory T-cell. (Created with BioRender.com)

Single-cell sequencing technologies such as single-cell mRNA sequencing (scRNA-seq) offer a high-throughput, unbiased method to profile cell identity, state, and function at the transcriptomic level. This high resolution technique has enabled the identification of rare cell populations that are functionally important and contribute towards TME heterogeneity [31, 32]. Genome-level sequencing like scRNA-seq has allowed for additional analyses, such as inference of copy number variation, developmental trajectories, and gene regulatory networks (GRNs) [33]. scRNA-seq provides valuable insight in uncovering complexities in cancer and revealing intratumoral and intertumoral heterogeneity across patients that can affect disease response [34]. This is especially pertinent in the immune microenvironment, which contains diverse populations of immune cells, and whose dynamic properties influence disease response. scRNA-seq can be used to profile immune populations following ICI treatment, generate predictive biomarkers of resistance or response, assess immune effector function, and identify potential immune checkpoints. Here, we review single-cell transcriptomic research investigating ICI response in melanoma, breast cancer (BRCA), and pancreatic ductal adenocarcinoma (PDAC; Fig. 1, Table 1). These cancers reflect a spectrum of response to ICI, with roughly 50% of metastatic melanoma patients responding, nearly 20% of BRCA patients responsive, and the majority of PDAC patients as non-responders to anti-PD-1 immunotherapy [35–37].

**Table 1:** Selected single-cell RNA sequencing studies in melanoma, breast cancer, and pancreatic ductal adenocarcinoma

Cancer Type	Treatment	Number of Patients & Samples	Number of cells	Cell type of focus	Takeaway	Single-cell platform	Citation
Metastatic melanoma	None	19 patients (10 metastases to lymphoid tissues, 8 to distant sites, 1 primary acral melanoma)	4,645	Malignant cells, T cells	Malignant cells displayed transcriptional heterogeneity, with differential expression of genes associated with cell cycle, spatial arrangement, and drug-resistance. Variability in exhaustion programs, T cell activation, and clonal expansion amongst tumor-infiltrating T cells across untreated and treated patients was observed	SMART-Seq (Illumina)	
Melanoma	anti-PD-, anti-CTLA4	31 patients; 33 samples (15 post-ICI resistant)	7,186	T cells	Identified resistance program in malignant cells marked by T cell exclusion prior to treatment. This program correlated with “cold” niches, or regions with decreased T cell infiltration. Genes involved in the cell cycle, specifically CDK4/6, repressed this resistance program in malignant cells and sensitized melanoma tumors to ICI in mouse models	10 × Genomics Chromium	Jerby- Anon et al. [13]
Metastatic melanoma	anti-PD-1, anti-CTLA4	48 samples taken from 32 patients treated with ICI (35 anti-PD-1, 11 anti-CTLA4 + PD-1, and 2 anti-CTLA4 samples)	16,291	CD8 + T cells	Transcription factor TCF7 in CD8 + T cells is associated with favorable prognosis and has increased expression in responders	SMART-Seq (Illumina)	Sade-Feldman et al. [42]
Acral and subcutaneous melanoma	None	6 patients; 8 samples (5 acral, 3 subcutaneous)	63,394	CD8 + T cells, Tregs	TGFβ signaling, Type I IFN, and cholesterol efflux associated with favorable prognosis. Acral melanoma displayed decreased CD8 + T cells, increased CD8 + T cell exhaustion, and Treg enrichment relative to cutaneous melanoma	Chromium Next GEM Single Cell 5’ Kit v2 (10 × Genomics)	Zhang et al. [39]
Hormone-receptor (HR +) and triple-negative breast cancer (TNBC)	Pembrolizumab (anti-PD-1)	29 patients with ICI only and 11 patients treated with neoadjuvant chemotherapy prior to ICI	175,942	T cell expansion	A third of tumors with PD1-expressing T cells underwent clonal expansion upon anti-PD-1 treatment. Increased CD8 + T cells with cytotoxic activity and CD4 + T cells with Th1-associated (Ifnɣ) and follicular-helper T cell-marker expression	Chromium 3’-scRNA-seq Kit (10 × Genomics)	
Breast cancer (lymph node metastasis)	None	15 paired samples of primary tumors and axillary lymph nodes	96,796	Breast cancer stem cells	BCSCs (CD44, ALDH2, and ALDH6A1 +) displayed similar copy number variants as normal breast tissue. BCSCs evolved in order to metastasize to the lymph node, where they displayed NECTIN2-TIGIT -mediated interactions with their microenvironment that aided in immune escape and metastatic outgrowth upon colonization	GEXSCOPE Single Cell RNAseq Library Kit, (Singleron Biotechnologies)	Xu et al. [40]
TNBC	Atezolizumab (anti-PD-L1) + paclitaxel; paclitaxel alone	22 patients, half treated with atezolizumab + paclitaxel, remaining with paclitaxel alone	489,490	T cells, myeloid cells, B cells	CXCL13 + T cells, involved in activating a pro-inflammatory macrophage (M1-like) signature, increased with combination therapy. cDC1s, follicular B cells, and lymphoid tissue inducer cells all increase following combination therapy, but decrease with paclitaxel treatment alone	Chromium Next GEM Single Cell 5’ Kit v2 (10 × Genomics)	Zhang et al. [43]
Local and metastatic TNBC	anti-PD1 + various chemotherapies	3 mice tumors pooled for each treatment level (8)	At least 5,000 cells per condition	T cells, macrophages	Decreased exhausted T cells and Tregs following anti-PD-1 + chemotherapeutic treatment. Decreased in M2-like + increase in M1-like macrophages	Chromium Next GEM Single Cell 5’ Kit v2 and v3 (10 × Genomics)	Carpen et al. [45]
PDAC	anti-PD-1	9 tumors from untreated PDAC patients (four dense-type PDACs, three loose-type, and two moderate-type)	77,121 (38,831 with 38,290 from public data)	Cancer- associated fibroblasts (CAFs)	CAF subtype with a highly-activated metabolic state (meCAFs) was found in looser-stroma PDAC tumors compared to tumors with denser stroma. Patients with high meCAF levels had a higher risk of metastasis and worse prognosis, but displayed a significantly better response to anti-PD-1 immunotherapy	Chromium Next GEM Single Cell 5’ Kit v2 (10 × Genomics)	
PDAC	anti-PD-1	Four murine models of PDAC were established using different pancreatic cancer cell lines	9056 (4470 from Panc02-tumors, 4586 from Panc02-H7 tumors)	CD8 + T cells, M2-like macrophages	Panc02-H7 tumors resistant to ICI had increased CD8 + T cell exhaustion and M2-like macrophages. M2-like macrophages and exhausted effector CD8 + T cells were key in predicting response to anti-PD-1 ICI in PDAC	Chromium Next GEM Single Cell Kit (10 × Genomics)	
PDAC	None	6 PDAC and 6 adjacent noncancerous tissues	58,076 (32,849 from PDAC tissues 25,227 from non-cancerous)	T cells, macrophages	Revealed low PD-L1 expression in PDAC. CCL5/SDC-1 receptor-ligand interactions were also present, and are known to promote crosstalk between T cells and malignant cells. Low PD-L1 expression may illuminate the lack of responsiveness to ICI in PDAC	Chromium Next GEM Chip G Single Cell Kit (10 × Genomics)	

PDAC: Pancreatic ductal adenocarcinoma; TNBC: Triple-negative breast cancer; BCSC: Breast cancer stem cell

### Profiling tumor heterogeneity and the immune microenvironment in a pre-treatment setting

Various studies have profiled the TME, gaining a deeper understanding of tumor heterogeneity and of the diverse immune cell populations present within the TME. A groundbreaking study used scRNA-seq to profile heterogeneity in metastatic melanoma [38]. 4645 single cells, including tumor, immune, and stromal cells, were isolated from 19 melanoma patients, and included samples from metastases to lymphoid tissues, intramuscular tissue, and the gastrointestinal tract, alongside one primary acral melanoma tumor sample. Within a tumor, malignant cells displayed transcriptional heterogeneity, with differential expression of genes associated with cell cycle, spatial arrangement, and drug-resistance. All tumors characterized by high levels of the MITF transcription factor also contained cells with low MITF and elevated levels of the AXL kinase, indicative of intratumoral heterogeneity. The single-cell expression patterns of 2,068 T cells from 15 treatment-naive melanomas were also analyzed, revealing variability in exhaustion programs, T cell activation, and clonal expansion amongst tumor-infiltrating T cells across patients. Overall, this work sheds light on the promise of scRNA-seq on profiling tumoral heterogeneity, identifying transcriptional programs within malignant or immune cells, and gaining a deeper understanding of immune function within the TME, with implications for immune therapies. Recent work utilized scRNA-seq to elucidate distinguishing factors between melanoma subtypes with differing prognoses [39]. This work used 8 melanoma samples (5 acral melanoma samples and 3 cutaneous melanoma) obtained from 6 melanoma patients. Of the 8 samples, 7 primary samples and one metastatic sample with metastasis to the lymph node were utilized. Upon scRNA-seq analysis, 5 functional cell clusters were defined and their association with melanoma prognosis determined. TGF-β signaling, Type I interferon activity, and cholesterol efflux related clusters were all associated with favorable prognosis in melanoma. Compared to cutaneous melanoma, acral melanoma samples displayed an immunosuppressive environment associated with decreased cytotoxic CD8 + T cells, increased CD8 + T cell exhaustion, and enrichment of immunosuppressive Tregs. This work provides insight into heterogeneity and differential signaling pathways within melanoma subtypes—factors that can serve as indicators of prognosis [39].

scRNA-seq has not only been used to profile the TME, but interactions of tumor cells with their microenvironment and the roles of these tumor cells in metastasis. 15 paired samples of breast primary tumors and breast cancer tumors that metastasized to axillary lymph nodes without any treatment were sequenced [40]. Nine clusters within cancer cells were identified, including breast cancer stem cells (BCSCs), denoted as CD44, ALDH2, and ALDH6A1 + cells. These BCSCs existed in primary tumors and displayed similar copy number variants as normal breast tissue. Strikingly, the BCSCs evolved in order to metastasize to the lymph node, where they displayed NECTIN2-TIGIT-mediated interactions with their microenvironment that aided in immune escape and metastatic outgrowth upon colonization. While several genes were upregulated within the metastatic samples, substantial intratumoral and intertumoral heterogeneity was present, and further corroborated with the notion that lymph node metastases within breast cancer contribute significantly to the aggressive nature of the disease. The identification of various signaling networks and profiling of differences between primary tumors and breast cancer lymph node metastasis via scRNA-seq presents a key strategy to predict interactions of the tumor with its microenvironment. Moreover, this can be used to assess cellular activity and tumor heterogeneity that can lead to the metastatpancic dissemination that significantly worsens patient outcomes. Within pancreatic cancer, scRNA-seq was conducted on PDAC tumors and adjacent non-cancerous tissues to evaluate the immune landscape of PDAC and potential clinically-relevant immune features [41]. This analysis revealed low PD-L1 expression in PDAC. Low PD-L1 expression may illuminate the lack of responsiveness to immunotherapies like anti-PD-1 in the PDAC context (Fig. 1). CCL5/SDC-1 receptor-ligand interactions were also present, and are known to promote pro-tumor crosstalk between T cells and malignant cells.

### Identifying programs that promote response or resistance to immunotherapy

While nearly half of melanoma patients are responsive to ICIs, there remains a significant portion of patients that derive no clinical benefit [37]. Moreover, the molecular mechanisms underlying such resistance are not completely understood. scRNA-seq was used to investigate tumor cell states associated with tumor progression and immune evasion in 33 melanoma tumors treated with anti-PD-1 [13]. A resistance program in these malignant cells prior to anti-PD-1 treatment was identified. This program was marked by T cell exclusion and correlated with “cold” niches, or regions with decreased T cell infiltration, a factor often associated with patient survival. Genes involved in the cell cycle, specifically CDK4/6, were found to repress this resistance program in malignant cells and sensitize melanoma tumors to ICI in mouse models. This work reveals a molecular signature associated with immune evasion and resistance to ICI, alongside a strategy to overcome therapeutic resistance to ICI in melanomas [13]. Another study utilized scRNA-seq to determine factors that predict response to ICIs within melanoma [42]. 48 tumor samples from patients treated with ICIs (35 anti-PD-1; 11 anti-CTLA4 + PD-1; and 2 anti-CTLA4) were profiled and two clusters of CD8 + T cells, associated with tumor progression or regression respectively, were identified. A TF, TCF7, involved in T cell differentiation, was identified in responsive patients and increased relative to non-responsive patients. These patient samples also demonstrated increased anti-tumor immunity as a result of TCF7 expression, indicating the identification of a potential therapeutic immune predictor of response to ICIs in melanoma (Fig. 1).

In breast cancer, scRNA-seq was used to examine the immune microenvironment of 22 patients with advanced TNBC [43]. These patients were either treated with chemotherapy (paclitaxel) alone or in combination with anti-PD-1. Here, CXCL13 + T cells, whose signaling is involved in activating a pro-inflammatory macrophage (M1-like) signature, increased following combination therapy. In addition to increased CXCL13 + T cells, conventional type 1 dendritic cells (cDC1s), follicular B cells, and lymphoid tissue inducer cells all increased following combination therapy, but decreased with chemotherapeutic treatment alone. This indicates the therapeutic role of CXCL13 + T cells in mediating effector activity of other immune cells in TNBC (Fig. 1), as well as the promise of a combinatorial approach rather than chemotherapy alone, which could compromise patient outcomes [43]. Sequential approaches of immunotherapy prior to neoadjuvant chemotherapy have also been shown to improve complete response in breast cancer [35]. Single-cell sequencing was utilized to assess the molecular landscape of tumor biopsies in hormone receptor-positive and triple-negative breast cancer (TNBC) cancer patients treated with anti-PD-1 antibody prior to certain patients receiving neoadjuvant chemotherapy [44]. In pre-treatment biopsies, the relative frequency of PD-L1 + dendritic cells, CCR2 + or MMP9 + macrophages, and cancer cells exhibiting MHC class I/II expression correlated positively with T cell expansion. Conversely, undifferentiated pre-effector/memory T cells and inhibitory macrophages were inversely correlated with T cell expansion. Upon anti-PD-1 treatment, a third of tumors contained PD-1-expressing T cells underwent clonal expansion, including expansion of CD8 + T cells with cytotoxic activity and CD4 + T cells with Th1-associated (Ifn-ɣ) and follicular-helper T cell-marker expression. Here, various immunophenotypes and associated gene sets following anti-PD-1 treatment were identified, providing insight into heterogeneity in PD-1 response in breast cancer. The effects of combination therapy of ICI and chemotherapy were also explored in a murine model [45]. Syngeneic, immunocompetent mice with local or metastatic TNBC were treated with either various chemotherapies or anti-PD-1 ICI separately or in combination. scRNA-seq was used to profile the immune landscape to provide a complete picture of cell populations and potential effector activity following these respective treatments, with tumors from three mice pooled for each treatment condition. The most effective treatment in vivo was medium dosage cyclophosphamide (C140) plus vinorelbine and anti-PD-1. Compared to untreated mice, a decrease in exhausted T cells and regulatory T cells (Tregs) was present following treatment. In addition, M2-like (anti-inflammatory and pro-tumor) macrophages were enriched in ineffective treatments while M1-like (pro-inflammatory and anti-tumor) macrophage populations were enriched in effective treatment combinations (Fig. 1). Based on the chemotherapeutic agent used, certain cell populations were more active than others. For example, treatment with cisplatin increased B cell proliferation. Thus, this work surveyed the immune landscape of local and metastatic TNBC following treatment with both chemotherapy and ICI alone or in combination, providing insights into mechanisms of action for these therapeutics and course of treatment.

Within pancreatic cancer, while PDAC is known to be non-responsive to ICI, mechanisms of resistance remain unknown. Four murine models of PDAC were established using different pancreatic cancer cell lines [46]. Pac02-H7 PDAC cell xenografts responded to anti-PD-1 treatment, with significantly reduced tumor growth and increased survival. scRNA-seq was used to characterize the microenvironments of Panc02-H7, which consisted of anti-PD-1 responsive and non-responsive tumors. Panc02-H7 tumors resistant to ICI had increased CD8 + T cell exhaustion and M2-like macrophages. The increasingly important role of macrophages in conjunction with exhausted effector CD8 + T cells in predicting response to anti-PD-1 ICI in PDAC was illuminated. Cancer-associated fibroblasts (CAFs) are another key modulator of the stroma within the TME and can promote the growth and invasion of the tumor. scRNA-seq was used to investigate intertumoral heterogeneity between PDAC patients with varying levels of desmoplasia, a stroma associated with increased invasion [47]. While no difference in CAF abundance was detected among tumors with varying levels of desmoplasia, a CAF subtype with a highly-activated metabolic state (meCAFs) was found in looser-stroma PDAC tumors compared to tumors with denser stroma. Patients with high meCAF levels had a higher risk of metastasis and worse prognosis. However, these patients also displayed a significantly better response to immunotherapy. A novel CAF subtype involved in PDAC progression that also promotes susceptibility to immunotherapy was identified [47].

Thus, scRNA-seq offers a high-throughput method to uncover heterogeneity within tumors and their microenvironment, and can be used to identify novel immune populations, determine markers of resistance, and assess molecular changes following ICI treatment (Fig. 1, Table 1). The insights gained by scRNA-seq can be further amplified by spatial information using spatial transcriptomics.

## Spatial transcriptomics in cancer immunotherapy

As discussed above, single-cell RNA sequencing has empowered the study of gene expression at the individual cell level, and since then revolutionized many fields of biological studies. However, most scRNA-seq approaches require dissociation of cells from their tissues, resulting in loss of information about their relative positionings which are critical to determining cellular subtypes and states, neighboring structures, transcriptional patterns and regulatory functions. Spatially-resolved transcriptomics, or spatial transcriptomics, is a groundbreaking molecular profiling technique that measures mRNA expression with locational context. It enables us to not only measure but also map genetic activities within tissue samples. Because of the close range of paracrine signals [48], this also allows us to uncover important patterns of cell–cell communication. Most spatial transcriptomics technologies use either an imaging-based in situ approach or spatial indexing enabled by local hybridization of barcodes to RNA molecules. Multiplexed error-robust fluorescence in situ hybridization (MERFISH) is an imaging-based approach that brings high spatial resolution at the single-cell level and the ability to visualize gene expression patterns directly within tissue Sects.  [49, 50]. STARmap is another approach that combines hydrogel-tissue chemistry and in situ DNA sequencing to achieve intact-tissue single-cell measurement of expression of more than a thousand genes [51]. Spatial indexing approaches, such as Slide-seq V2 [52] and Visium Spatial Gene Expression by 10 × Genomics [53], enable the quantification of genome-wide expression with higher throughput and lower cost, although at a lower spatial resolution (10-micron and 55-micron, respectively). Xenium is a novel platform from 10 × Genomics that allows the characterization of RNAs and multiplexed proteins with subcellular in situ resolution [54]. Finally, NanoString GeoMx digital spatial profiler (DSP) captures at single-cell resolution and can be used for large-scale spatial profiling of targeted gene expression in tissue samples [55].

**Fig. 2:**
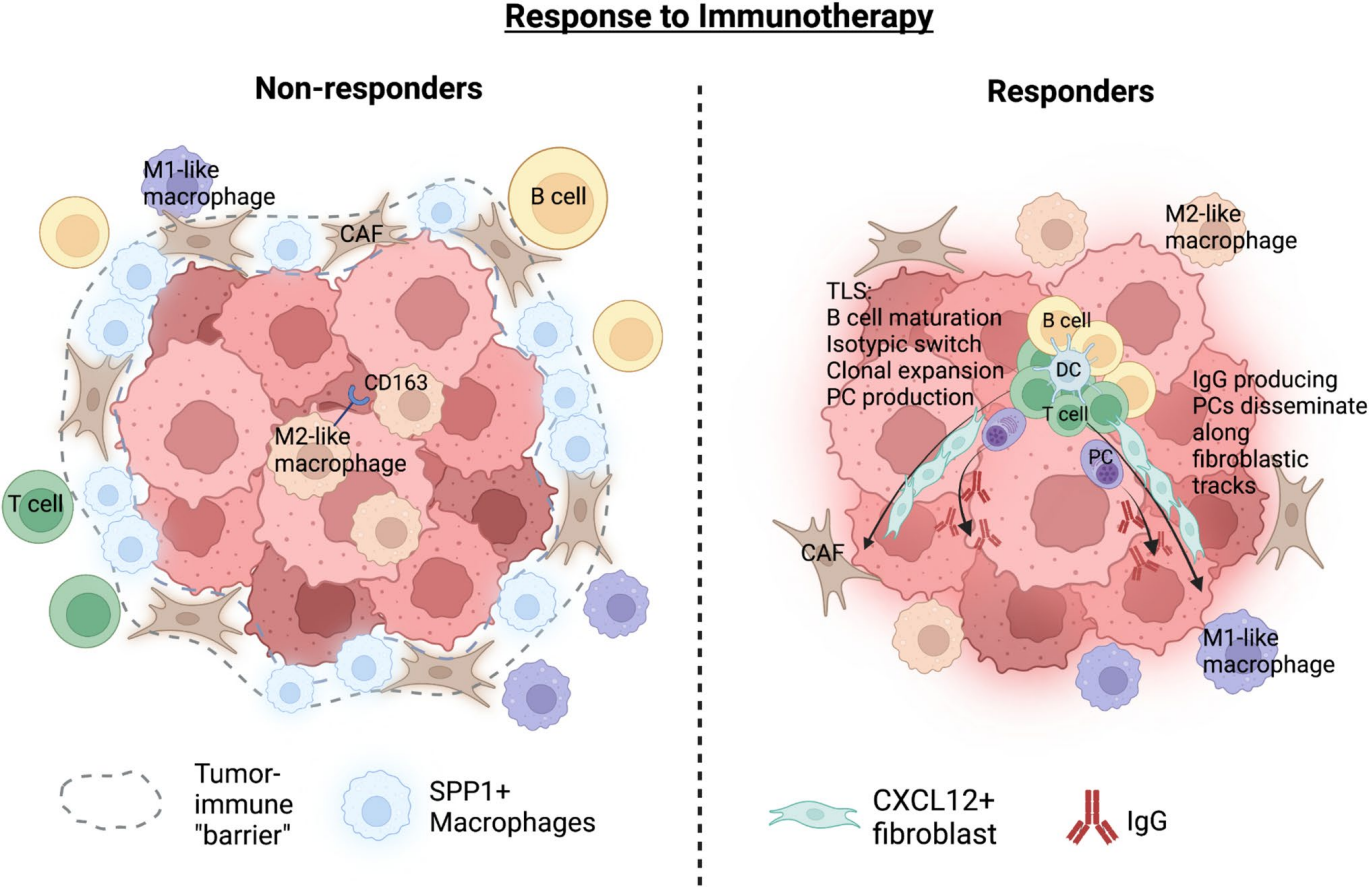
Spatial distribution and interactions of cells within the TME of ICI responders vs. non-responders. Left: High CD163 + intratumoral infiltration is associated with worse clinical outcomes in NSCLC [61]. A tumor immune “barrier” (TIB) structure in the HCC microenvironment leads to resistance to ICIs [68]. SPP1 + macrophages and CAFs interact to promote the formation of the TIB structure and limit immune infiltration of tumors. Right: Mature TLS in RCC patients responding to ICI mediates maturation of B cells, and allows for selection, clonal amplification and dissemination of IgG-producing PCs [65]. TME, tumor microenvironment; ICI, immune-checkpoint inhibitor; NSCLC, non-small cell lung cancer; HCC, hepatocellular carcinoma; CAF, cancer-associated fibroblasts; NSCLC, non-small cell lung cancer; TAM, tumor-associated macrophages; TLS, tertiary lymphoid structure; RCC, renal cell cancer; PC, plasma cell. (Created with BioRender.com)

Spatial transcriptomics has emerged as a promising tool in cancer immunology research owing to its ability to provide information about gene expression patterns in the context of the TME. Researchers could therefore study the mechanisms by which tumors adapt to their environment and evade the immune system in their native context. For example, spatial transcriptomics has shown promise in tracing tumor cell lineages in various cancer types [56]. It has also been used to identify how PDAC cells and expression programs are organized in distinct multicellular communities and to spatially define receptor–ligand interactions that are differentially correlated between untreated and treated tumors [57]. Another group conducted a longitudinal analysis of a series of tumor samples, either biopsied or autopsied, from a single melanoma patient [58]. They traced the evolution of tumor cell populations considering site-specific variances in tumor-immune interactions, which led to immunotherapy resistance. Developing effective cancer therapies requires not only the characterization of tumor cells, but also a thorough understanding of other cell types located at the tumor boundary. Although bulk and single-cell RNA sequencing methods have been employed, they are limited in providing spatial resolution, which is readily addressed by spatial transcriptomics. By employing spatial transcriptomics to analyze the TME, many labs have discovered new cell phenotypes, intratumoral structures, biomarkers and cell–cell interactions that contribute to response or resistance to ICI treatment, which is summarized below (Fig. 2, Table 2).

**Table 2:** Selected spatial transcriptomics studies on ICI treatment response

Cancer type	Treatment	Number of Patients/Samples	Takeaway	Platform	Citation
PDAC	FOLFIRINOX chemotherapy followed by multifraction conformal radiotherapy with concurrent capecitabine or fluorouracil. 7 patients received losartan, and/or nivolumab	22 tumors (untreated, n = 15; treated, n = 7)	Revealed three multicellular communities with distinct contributions from malignant, fibroblast and immune subtypes: classical, squamoid-basaloid and treatment enriched. Discovered spatially defined intercellular receptor–ligand interactions specifically enhanced in post-treatment residual disease,	NanoString GeoMx DSP	Hwang et al. [57]
Melanoma	Intralesional TLR9 agonist + anti-PD-1	1 patient, 37 samples (across 9 years)	Existence of a neural crest tumor population in melanoma immunotherapy resistance and site-specific differences in tumor-immune interactions	Tissue cyclic immunofluorescence	Liu et al. [58]
HCC	Cabozantinib + nivolumab	7 patients (non-responders, n = 3; responders, n = 5)	The TME of responding tumors was enriched for immune cells and CAFs with pro-inflammatory signaling relative to the non-responders. In a refractory sample, an immune rich region recapitulates the transcriptional profile observed across all responders tumors while the immune poor area is similar to non-responders	10 × Visium	Zhang et al. [59]
NSCLC	ICI	152 patients, 16 samples ( low macrophage infiltration, n = 8; high macrophage infiltration, n = 8)	Lower level of CD163 + TAM infiltration was associated with better survival, but only in the tumorous compartment and not in the stroma. CD27, CCL5, and ITGAM were the three most significantly upregulated genes in tumors characterized by a high level of TAM infiltration	NanoString GeoMx DSP	Larroquette et al. [61]
Soft-tissue sarcomas	Pembrolizumab + low-dose cyclophosphamide	6 tumors (objective response, n = 3; progressive disease as the best response, n = 3)	Upregulation of genes encoding chemokines involved in TLS formation in responder patients. TLSs from non-responder patients were significantly more enriched in Tregs. There is a strong correlation between infiltration by PCs expressing surface IgG and response to ICI	NanoString GeoMx DSP	Italiano et al. [64]
RCC	Nivolumab, Nivolumab + Ipilimumab or tyrosine kinase VEGFR inhibitors	24 primary tumor sections (frozen, n = 12; FFPE, n = 12)	TLSs are sites of in situ B cell maturation toward plasma cells. IgG + PCs disseminate into the tumor tissue along fibroblastic tracks. Patients with IgG-labeled tumor cells have high response rate to ICI and prolonged PFS	10 × Visium	Meylan et al. [65]
NSCLC	ICI	6 tumors (objective response, n = 3; progressive disease as the best response, n = 3)	Tumor-to-stroma ratio of infiltrating CD8 + cells is lower in tumor lesions enriched in FAP + αSMA + CAFs	NanoString GeoMx DSP	Peyraud et al. [66]
Medulloblastoma	Palbociclib	1 patient	A distinct tumor “interface” where the tumor cells contacted neighboring mouse brain tissue, consisting of abundant astrocytes and microglia, and continued to proliferate despite Palbociclib treatment	Unknown	Tan et al. [67]
HCC	Anti-PD-1	8 patients (non-responders, n = 5; responders, n = 3) and adjacent normal tissue sections (n = 3)	A tumor immune barrier (TIB) structure in the TME contributes to the efficacy of immunotherapy. SPP1 + macrophages and CAFs interact to promote the formation of the TIB structure and limit immune infiltration of tumors. Blockade of SPP1 or macrophage-specific deletion of Spp1 in mice can destroy the TIB structure and sensitize HCC to immunotherapy	10 × Visium	Liu et al. [68]
HCC	Cemiplimab or nivolumab	1 responder	Cellular triads harboring mregDC and CXCL13 + Th cells may be critical for promoting local differentiation of PD-1^hi^ progenitor CD8 T cells into potent effector-like CD8 T cells and that these interactions in the TME are essential for the success of PD-1 blockade in HCC	MERFISH	Magen et al. [69]
NSCLC	ICI	21 patients (33 tumor regions of interest (ROI) and 23 stroma ROIs)	Patients responsive to ICI therapy expressed higher levels of IL2 receptor alpha (CD25) within their tumor compartments, which corresponded to increased IL2 mRNA within their stroma. Tumor expression of CD44 was depleted in the responsive patients, with higher stromal expression of SPP1 and improved prognosis	NanoString GeoMx DSP	Monkman et al. [70]
NSCLC	ICI	56 patients (2 distinct tumor TMA spots per patient)	A small set of candidate genes in the PanCK, CD45 and CD68 compartment that are associated with outcome	NanoString GeoMx DSP	Moutafi et al. [74]
Gastric cancer	ICI	12 tumor samples (responders, n = 5; non-responders, n = 7)	CXCL12-CXCR4, CD74-MIF, and LGALS9-LRP1 ligand-receptor interactions are stronger in non-responders	NanoString GeoMx DSP	Park et al. [75]

PDAC: Pancreatic ductal adenocarcinoma; DSP: Digital Spatial Profiler; HCC: Hepatocellular carcinoma; TME: Tumor microenvironment; CAF: Cancer-associated fibroblasts; NSCLC: Non-small cell lung cancer; ICI: Immune-checkpoint inhibitor; TAM: Tumor-associated macrophages; TLS: Tertiary lymphoid structure; Treg: Regulatory T cell; PC: Plasma cell; RCC: Renal cell cancer; PFS: Prognosis-free survival; MERFISH: Multiplexed error-robust fluorescence in situ hybridization; IL2: Interleukin-2

### Mapping immune spatial distribution and intratumoral sub-structures

Spatial transcriptomics has made it possible to preserve heterogeneity in biopsies that cannot be detected by conventional transcriptome analysis. We could visualize the immune cell composition and spatial distribution differences between the tumor and stromal compartments, and infer their correlations with clinical responses [59]. For example, tumor-associated macrophages (TAMs) are one of the most abundant immune cells in the TME. Intratumoral macrophage infiltration levels have been proposed as an indicator of ICI resistance [60]. Moreover, the distance between tumor cells and TAMs may also play an important role in assessing the prognostic impact based on spatial transcriptomics. In one study, researchers validated that high intratumoral macrophage infiltration was associated with poor clinical outcomes of non-small cell lung cancer (NSCLS) patients [61](Fig. 2). However, CD163 infiltration in the stroma compartment further away from the tumor was not significantly associated with survival, confirming the importance of spatial distribution of immune cells specifically in the TME. This aligns with previous findings on longer survival of patients with tumor cells that were distant from TAMs [62]. They then used the NanoString GeoMx Immune Pathways assay to examine both tumorous and stromal compartments. Comparative spatial analysis suggested that upregulation of ITGAM, CCL5 and CD27 and downregulation of BCL2 and HLA-E might be involved in the homing of macrophage within the tumor. Despite a negative correlation between HLA-E and CD163 infiltration level, results obtained through bulk RNAseq depicted an opposite trend, thus suggesting the value of spatial profiling to correlate gene expression with TME features. Finally, within patients with highly macrophages-infiltrated tumors, genes associated with M1-like (pro-inflammatory and anti-tumor) signatures and the IFN-γ signaling pathway were correlated with better responses under immunotherapy.

Additionally, in many different cancer types, the presence of tertiary lymphoid structures (TLSs) correlates with favorable prognosis [63]. One study revealed an upregulation of genes encoding chemokines involved in TLS formation in soft-tissue sarcoma patients showing ICI response [64]. They also confirmed that TLSs from non-responder patients were significantly more enriched in Tregs, and that infiltration by plasma cells (PCs) expressing surface IgG strongly correlated with response to ICI. Another group sought to use spatial transcriptomics to better understand the function of TLS by defining its role in situ within the TME [65]. Through classifying B-cell phenotypes and localizing them within renal cell carcinoma (RCC) patient samples, they suggested that TLSs serve as sites for maturation, clonal amplification and isotype switching of B cells and for harboring PCs before they disseminate along CXCL12-positive fibroblastic cell tracks (Fig. 2). In addition, TLS + tumors exhibited more frequent antibody production which correlated with an increase in therapeutic responses and PFS in ICI-treated RCC patients. A more recent study investigated NSCLC tumor lesions enriched in FAP + αSMA + CAFs, where a lower level of infiltrating CD8 + cells was uncovered [66]. Overall response rate and survival of patients with TLS-positive tumors enriched in this subset of CAFs were significantly lower than those of patients with low abundance of these CAFs [66]. This implies that development of novel CAF-targeting therapies could be beneficial in this patient population.

Recent works have also explored novel sub-structures in the TME that may determine the efficacy of ICI treatment. In mouse models, for example, a distinct tumor interface has been identified where the tumor cells contacted neighboring brain tissue and continued to proliferate despite Palbociclib treatment [67]. Using a combination of spatial transcriptomics, scRNA-seq and multiplexed immunofluorescence, the authors identified a spatial niche of SPP1 + macrophages and CAFs located near the tumor boundary of hepatocellular carcinoma (HCC) patient [68]. They found that these clusters formed a tumor immune “barrier” in non-responders, but not in responders to ICI treatment (Fig. 2). They further demonstrated that inhibition of SPP1 or macrophage-specific deletion of SPP1 in mice led to enhanced efficacy of anti-PD-1 treatment in liver cancer. In a different study, investigators observed local expansion of CXCL13^+^ Th and PD-1^hi^ effector-like CD8 T cells in renal cell cancer (RCC) responders, and searched for local interactions that underpin these changes [69]. Probing the spatial interactions between these T cells and a DC state enriched in maturation and regulatory markers (mregDC, reviewed in our back-to-back review [28]) with MERFISH, they found clusters of progenitor-resembling T cells, CXCL13 + Th cells, and mregDCs that co-localized in discrete regions that were populated by B cells in an ICI responder. Therefore, they hypothesized that direct interactions between these cells were important for effective T cell responses, and that these niches promoted the local differentiation of PD-1^hi^ progenitor CD8 T cells into potent effector-like CD8 + T cells.

### Elucidating understudied immune functions and novel biomarkers

Spatially resolved data also provides new or alternative evidence of immune and cytokine functions that remain understudied. One study used DSP to analyze tumor and TME compartments from a cohort of NSCLC patients treated with ICI [70]. Differential protein marker analysis performed between responders and non-responders indicated that EPCAM was enriched in the stroma of responding patients. This finding contrasts a previous hypothesis that the presence of EPCAM expression is associated with more aggressive prostate cancer [71], possibly due to differences in cancer types and the technologies used (standard immunohistochemistry vs. DSP). Validating DSP counts with multiplex immunofluorescence, they then identified an Interleukin-2 (IL2) axis between tumor and stroma compartments, and hence IL2 receptor alpha (CD25) as a potential predictive biomarker of response. The positive correlation between IL2 mRNA and pro-apoptotic markers suggested that endogenous levels of this cytokine were key to recruiting and sustaining the prerequisite cell phenotypes for ICI efficacy. Moreover, tumor expression of CD44, a hallmark of cancer cell “stemness” that promotes tumor survival and proliferation [72], was upregulated in refractory patients and depleted in the responsive ones, with higher stromal expression of one of its ligands, SPP1, in the responsive group*.* Interestingly, SPP1 had previously been implicated as both an immune suppressor and enhancer [68, 73]. Results from this study aligned with the latter, and suggested that SPP1 may have an important role in generating an ICI-sensitive niche in tumors. This further underscores how data derived from spatial transcriptomics prompts us to reassess the role of key markers. By profiling different molecularly defined compartments for tumor cells and the TME using spatial transcriptomics, researchers have also uncovered novel biomarkers that are likely to be important for treatment efficacy. Several genes in the tumor (panCK +), CD45 and CD68 compartments have been shown to predict survival from a recent study [74]. Certain ligand-receptor interactions have been found to be stronger in non-responders to ICI, including CXCL12-CXCR4, CD74-MIF, and LGALS9-LRP1 [75]. These findings suggest that the interaction between tumor cells and their adjacent stromal and immune cells within the TME may also influence the response to ICIs.

### Integration of spatial transcriptomics and single-cell RNA sequencing data

Despite the rapid advancement in spatial transcriptomic methods, resolution limitations remain a challenge. Moreover, meaningfully interpreting these results requires new approaches and analysis methods. Many studies have demonstrated the benefits of decomposing spatially resolved data by integrating spatial and single-cell transcriptomics data. For example, one study used spatial barcoding and scRNA-seq to locate an immunosuppressive tumor-specific keratinocyte subpopulation to a fibrovascular niche at the tumor borders in squamous cell carcinoma samples [76]. Two primary approaches for this integration are deconvolution and mapping. Deconvolution aims to characterize the proportion of each cell type in each spot, while mapping assigns a dominant cell type to each spot. Two recent studies benchmarked integration methods of scRNA-seq and spatial transcriptomics [77, 78]. Mapping-based methods, such as Seurat [79]and MIA [80], transfer probabilistic annotations from a reference (scRNA-seq) to a query set (spatial data). Deconvolution methods, such as Cell2Location [81], CARD [77], spatialDWLS [82] and RCTD [83], rely on probabilistic models to infer cell type proportions of each spot. Deep learning frameworks have also quickly emerged in this field. For example, gimVI uses a generative model to predict spatial location of undetected transcripts [84]. Tangram combines non-convex optimization and deep learning to learn the spatial alignment for scRNA-seq data [85]. Finally, reference-free methods, such as STdeconvolve [86], have also been proposed for deciphering spatial patterns using locations of spots and their gene expression profiles.

## Conclusion and future directions

In this review, we have provided an overview of the current state-of-the-art understanding of the TME and its molecular and cellular landscape dynamics in response to ICI treatments. Additionally, we have highlighted the significance of scRNA-seq and spatial transcriptomics as powerful technologies that have advanced this field. Through the studies discussed, these techniques have yielded valuable insights into immune cell development, their interactions with the TME, and their influence on tumor progression and response to ICI. By enabling high-definition mappings at the single-cell level, these methods offer the potential for more advanced inference of gene regulation and signaling networks within the TME.

Given the high economic costs and experimental complexity, the number of tissues processed by scRNA-seq and spatial transcriptomics still remain limited, as evidenced by the relatively modest sample sizes of some of the studies discussed. Analysis on larger clinical cohorts are essential to corroborate these findings, especially considering the heterogeneous nature of cancer. Expanded use and continuous advancements of single-cell and spatial transcriptomics methods would be essential to identify alternative therapeutic interventions for cancer types that currently do not benefit from ICI treatments.

Recent innovations have significantly increased the resolution of spatial transcriptomics. For example, the Xenium platform enables single cell spatial imaging of thousands of RNAs and offers the integration of gene expression with histological images in the same tissue section  [87]. For scRNA-seq, newer methods of cell dissociation and greater sequencing depth have enabled higher resolution profiling of the TME; however, these methods are still being refined to capture low-abundance transcripts. Concurrently, numerous analytical methods are being developed to extract meaningful features from this wealth of high-dimensional data. These advancements in technologies and methods provide a solid foundation for a novel, integrative, and spatially defined transcriptomic approach to discover novel biomarkers for immunotherapy and ultimately pave the way for the development of effective and personalized cancer therapies.
